# Fulminant hepatitis B reactivation leading to liver transplantation in a patient with chronic hepatitis C treated with simeprevir and sofosbuvir: a case report

**DOI:** 10.1186/s13256-015-0630-8

**Published:** 2015-07-28

**Authors:** Alexander R. Ende, Nina H. Kim, Matthew M. Yeh, Jason Harper, Charles S. Landis

**Affiliations:** 1 Department of Medicine, Division of Gastroenterology and Hepatology, University of Washington, 1959 Northeast Pacific Street, Seattle, WA 98195 USA; 2 Department of Medicine, Division of Infectious Disease, University of Washington, 1959 Northeast Pacific Street, Seattle, WA 98195 USA; 3 Department of Pathology, University of Washington, 1959 Northeast Pacific Street, Seattle, WA 98195 USA

**Keywords:** Hepatitis B, Hepatitis C, Simeprevir, Sofosbuvir, Fulminant liver failure, Liver transplant, Hepatitis B reactivation

## Abstract

**Introduction:**

Hepatitis B and C coinfection is commonly seen in clinical practice. In coinfected individuals, high levels of hepatitis C viremia are often associated with low levels of serum hepatitis B DNA. Hepatitis B reactivation in hepatitis C-infected patients treated with pegylated interferon and ribavirin has been reported, but severe or fulminant reactivation is uncommon. Hepatitis C treatment-associated hepatitis B reactivation in patients with chronic hepatitis C and isolated core antibody has not been reported previously.

**Case presentation:**

A 59-year-old white woman with chronic hepatitis C genotype 1B and isolated hepatitis B core antibody initiated treatment with simeprevir, sofosbuvir, and ribavirin for treatment of chronic hepatitis C. She responded very well to treatment initially with near normalization of aminotransferases and hepatitis C viral load suppressed to below the level of quantification after 4 weeks of treatment. At week 11 of a planned 12-week course, she developed fulminant hepatic failure due to hepatitis B reactivation and ultimately required liver transplantation. Fortunately, her posttransplant clinical course was unremarkable.

**Conclusions:**

This is the first report of hepatitis B reactivation in a patient with isolated hepatitis B core antibody leading to fulminant hepatic failure and liver transplantation after initiation of treatment with sofosbuvir, simeprevir, and ribavirin for hepatitis C. This case raises the concern for the risk of severe hepatitis B reactivation in hepatitis B and C-coinfected patients or chronic hepatitis C-infected patients with isolated hepatitis B core antibody treated with direct-acting antiviral drugs for hepatitis C.

## Introduction

Sofosbuvir and simeprevir were approved by the Food and Drug Administration (FDA) for treatment of chronic hepatitis C virus (HCV) in 2013. Sofosbuvir is a novel nucleotide analogue inhibitor of the hepatitis C virus NS5B polymerase with potent antiviral activity. Simeprevir is a second-generation inhibitor of the hepatitis C NS3/4A protease. The combination of these agents for treatment of chronic hepatitis was rapidly adopted because of improved safety, tolerability and efficacy compared with prior pegylated interferon (Peg-IFN)-based treatments [1].

A large proportion of HCV-infected patients, as high as 61.5% in one study [2], have evidence of prior exposure to hepatitis B virus (HBV) with the profile of an isolated core antibody [2–6]. Here we report the first case of HBV reactivation in a patient with isolated HBV core antibody (HBcAb) leading to fulminant hepatic failure and liver transplantation following treatment with simeprevir, sofosbuvir, and ribavirin for HCV.

## Case presentation

**Fig. 1:**
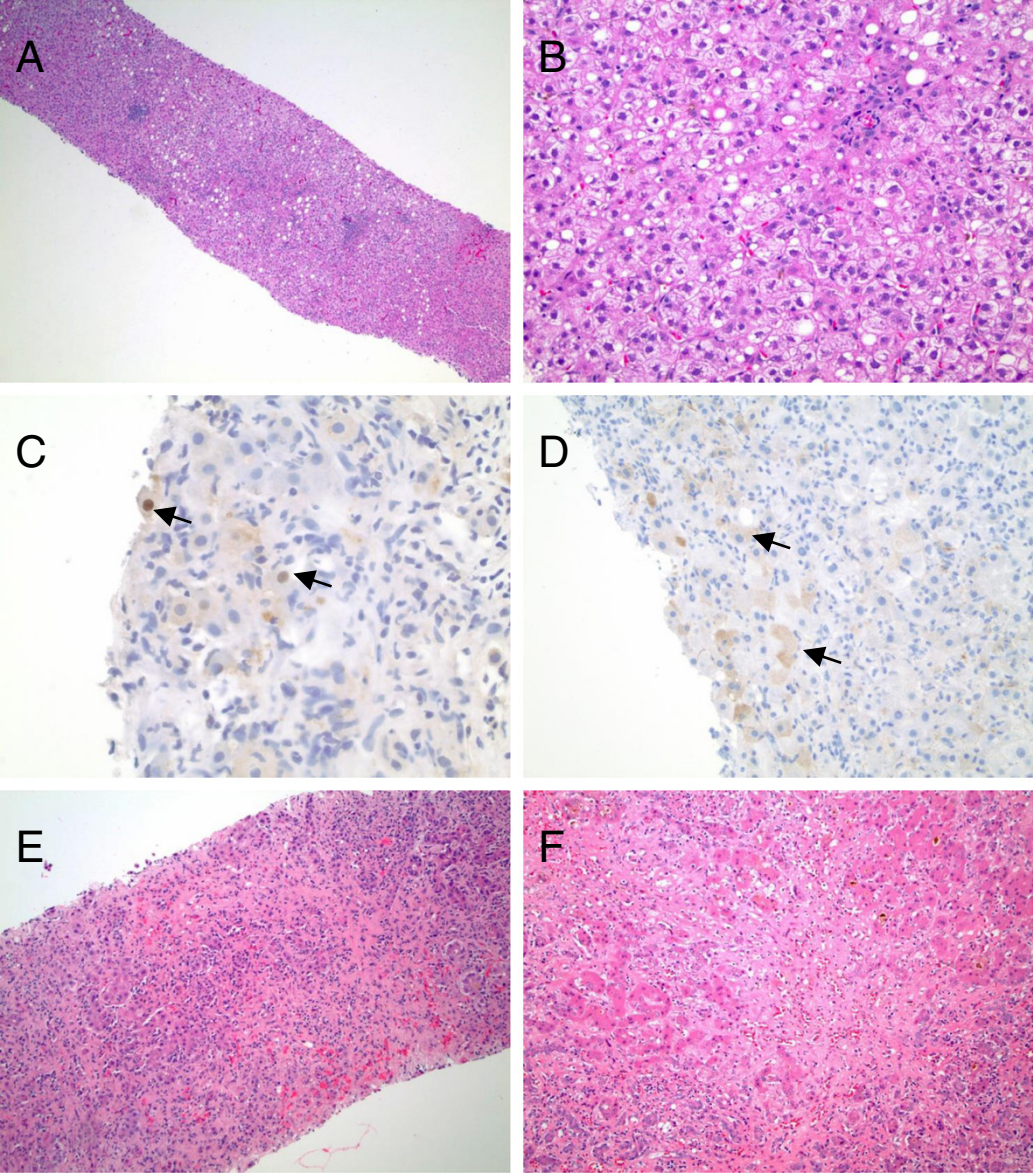
**a** Low-power magnification of a biopsy specimen from 8 months prior to presentation shows chronic hepatitis with mild steatosis, and mild portal and lobular inflammation. **b** High-power magnification of specimen in (**a**) demonstrates the component of steatohepatitis, composed of areas of steatosis, ballooned hepatocytes, and lobular inflammation. **c** and **d** Diagnostic core needle biopsy performed one week before liver transplantation shows hepatocytes with positive immunohistochemical nuclear staining for hepatitis B virus core antigen (**c**) and positive immunohistochemical cytoplasmic staining for hepatitis B virus surface antigen (**d**). Arrows in (**c**) and (**d**) indicate examples of positive immunohistochemical staining. Immunohistochemistry for hepatitis B virus surface antigen was negative on a liver biopsy specimen obtained 8 months prior to presentation (not shown). **e** Core needle biopsy performed 1 week before liver transplantation shows severe hepatitis with bridging and confluent necrosis, marked inflammation, and ductular reaction. **f** High-magnification view of liver explant specimen shows confluent necrosis and bridging necrosis with bile ductular reaction and cholestasis

A 59-year-old white woman with chronic HCV genotype 1B had initiated treatment with simeprevir, sofosbuvir, and ribavirin. She had been treated with standard interferon and ribavirin in the remote past with no response. She also had a history of Burkitt’s lymphoma in remission for 2 years. Her pretreatment laboratory test results were remarkable for aspartate aminotransferase (AST) 235 and alanine transaminase (ALT) 168U/L. She had a liver biopsy 8 months prior to presentation showing grade 1 inflammation and stage 2/4 fibrosis with histopathologic features of both chronic HCV and nonalcoholic steatohepatitis (Fig. 1a, b). Immunohistochemistry for HBV surface and core antigens in her liver biopsy specimen were negative. Her serum HBV surface antigen (HBsAg) was nonreactive at that time with reactive core antibody but absent surface antibody. A HBV deoxyribonucleic acid (DNA) level measured 2 years prior to this presentation was undetectable. Her 4-week HCV level was less than 12IU/mL with AST and ALT approaching normal at 38 and 31U/L, respectively. Her ribavirin was discontinued at that time because of anemia.

At week 11 of a planned 12-week course, she was found to have ALT 2263U/L, AST 2870U/L, total bilirubin of 9.1mg/dL and an international normalized ratio (INR) of 1.9. Repeat HBV serologies revealed a positive HBsAg and a viral load of 29,000,000IU/mL. Her HCV viral load was undetectable. A transjugular liver biopsy revealed severe hepatitis with confluent necrosis, marked portal and lobular inflammation, many apoptotic bodies, and extensive periportal and pericellular fibrosis (Fig. 1e). HBV surface and core antigens were identified on immunohistochemistry (Fig. 1c, d).

Tenofovir was started on day 3 of presentation for treatment of HBV. Despite treatment, she became increasingly encephalopathic with a rising INR and was transferred to the intensive care unit. She underwent liver transplantation 10 days into her hospital admission. Her posttransplant course was unremarkable and she was discharged home 1 week posttransplant. The liver explant pathology showed an area of confluent necrosis with extensive reticular collapse with no diagnostic evidence of lymphoma (Fig. 1f). Her HCV viral load was tested at 12 and 24 weeks posttransplant and has remained undetectable. HBV remains suppressed with ongoing tenofovir therapy.

## Discussion

Approximately 350 million people are infected with HBV and 170 million people are infected with HCV worldwide. The exact number of patients infected with both HCV and HBV is unknown, but estimates of the rates of HCV coinfection in individuals with HBV (HBsAg positive) vary from 9 to 30 %, depending on geographic region [7]. The incidence of isolated HBcAb in HCV-infected individuals is likely even higher because screening programs often only test for HBsAg [2, 3].

Several studies have shown that the HBV and HCV interact with each other and HCV infection can suppress HBV replication [2]. Coinfected patients commonly have lower HBV DNA levels, and decreased hepatic expression of HBsAg and HBV core antigen compared to HBV monoinfected patients [2–7]. In addition, in vitro studies have also shown evidence that HCV can suppress HBV replication [2]. This has led to the hypothesis of reciprocal interaction between viruses where HBV replication is inhibited by HCV. Hence, the potential for reactivation of HBV following HCV treatment has long been a concern.

We are not aware of any data or reports of HBV reactivation with the recently approved direct-acting antivirals for HCV. However, HBV reactivation with Peg-IFN and ribavirin treatment has been reported in patients chronically coinfected with HBV and HCV. One report documented the reappearance of HBV DNA in 38% of HBV/HCV-coinfected patients during Peg-IFN/ribavirin treatment [8]. However, no clinical hepatitis developed and HBsAg seroconversion occurred in 30% of patients, suggesting that interferon-based therapy suppresses both viruses. We are not aware of any studies describing treatment-associated HBV reactivation in patients with chronic HCV and isolated HBcAb.

Both Peg-IFN and ribavirin have been shown to be effective treatments for chronic HBV infection [8–10] and Peg-IFN is currently an approved treatment for chronic active HBV infection [9]. Until recently, standard of care for HCV treatment included both Peg-IFN and ribavirin. However, newly approved direct-acting antivirals, such as simeprevir and sofosbuvir are specifically directed to inhibit HCV replication and can be used in combination without Peg-IFN [1]. These new agents, unlike Peg-IFN and ribavirin, should not have any inhibitory effect on HBV. Therefore, using these agents in the absence of Peg-IFN may bring about increased risk for HBV reactivation in HCV/HBV-coinfected patients. Furthermore, we reason that this case represents evidence to support the theory of reciprocal HCV/HBV interaction most frequently characterized by an inhibition of HBV replication by HCV. With the potential inhibitory effect of chronic HCV on HBV eliminated with oral antiviral treatment in our patient, HBV replication was no longer restricted leading to fulminant HBV reactivation necessitating liver transplantation.

## Conclusions

This case raises concern for the safety of new direct-acting antiviral drugs in patients coinfected with HBV and HCV, or in those who have chronic HCV infection and isolated HBcAb. It remains unclear whether HBV booster immunization may have prevented this episode [11]. Close monitoring of aminotransferases and/or HBsAg or HBV DNA levels may be warranted in such patients during oral interferon-free direct-acting antiviral therapy.

## Consent

Written informed consent was obtained from the patient for publication of this case report and any accompanying images. A copy of the written consent is available for review by the Editor-in-Chief of this journal.

## Abbreviations

ALT: Alanine transaminase
AST: Aspartate aminotransferase
DNA: Deoxyribonucleic acid
FDA: Food and Drug Administration
Hb: Hemoglobin
HBcAb: HBV core antibody
HBsAb: HBV surface antibody
HBsAg: HBV surface antigen
HBV: Hepatitis B virus
HCV: Hepatitis C virus
INR: International normalized ratio
Peg-IFN: Pegylated interferon
RNA: Ribonucleic acid
